# Enhancing the Stability of Ag/CH_3_NH_3_PbBr_3_ Interfaces via AgBr Doping

**DOI:** 10.3390/nano16211379

**Published:** 2026-10-28

**Authors:** Chao Wang, Aiwu Yu, Yuzhu Pan, Yulian Zhang, Jinbao Chen, Xin Wang

**Affiliations:** 1 National Key Laboratory of Aerospace Mechanism, Nanjing University of Aeronautics and Astronautics, Nanjing 210016, China; 2 College of Physics, Nanjing University of Aeronautics and Astronautics, Nanjing 210016, China; 3 Shanghai Institute of Spacecraft Equipment, Shanghai 200240, China; 4 Shanghai Aerospace Equipments Manufacturer Co., Ltd., Shanghai 200245, China; 5 Key Laboratory of Semiconductor Display Materials and Chips, Suzhou Institute of Nano-Tech and Nano-Bionics, Chinese Academy of Sciences, Suzhou 215123, China

**Keywords:** electrode stability, MAPbBr_3_ single crystals, heterovalent doping, Ag electrode

## Abstract

While CH_3_NH_3_PbBr_3_ (MAPbBr_3_) single crystals exhibit exceptional potential for biased X/γ-ray detection, achieving a stable metal–semiconductor contact remains a critical challenge. Herein, we address this issue by incorporating AgBr as a dopant into pristine MAPbBr_3_ single crystals prior to Ag electrode deposition. Our results reveal a concentration-dependent interfacial mechanism: while low-level doping introduces excess Br^−^ ions and Pb vacancies that accelerate electrochemical corrosion, a high concentration of Ag^+^ within the lattice effectively suppresses the deleterious reaction between the Ag electrode and the perovskite. This work establishes a defect-engineering strategy to stabilize metal electrodes, offering a viable pathway for the development of reliable, high-performance MAPbBr_3_-based radiation detectors.

## 1. Introduction

CH_3_NH_3_PbBr_3_ (MAPbBr_3_) single crystals exhibit considerable promise for photon and particle detection across a wide energy range, encompassing X-rays, gamma-rays, visible light, protons, and alpha particles [1,2,3,4,5,6,7,8]. This applicability is underpinned by their balanced electron–hole mobility, tunable carrier type and concentration, and their exceptional tolerance to defects and irradiation [9,10].

A major challenge, however, lies in establishing stable metal–semiconductor contacts under high bias [11,12,13]. The external electric field triggers halide-ion migration through the lattice, which in turn promotes interfacial reactions with the electrodes and consequent device degradation. Even Au, widely presumed to be chemically inert, remains susceptible to such bias-induced reactions with MAPbBr_3_ [14,15,16]. Likewise, Ag electrodes, despite their lower work function, exhibit even more pronounced reactivity, rendering them unsuitable for this application [17,18].

MAPbBr_3_ single crystals are empirically observed to react with Ag electrodes even under zero bias, presenting a formidable obstacle to the fabrication of stable Ag/MAPbBr_3_ contacts [3,19]. To unravel the origin of this interfacial instability, we conducted a systematic investigation of the Ag/MAPbBr_3_ contact system.

In this work, MAPbBr_3_ single crystals incorporating varying AgBr doping concentrations were epitaxially grown on pristine MAPbBr_3_ substrates, followed by the deposition of two coplanar Ag electrodes [20]. Post-bias characterization revealed that at low doping levels (<15%), excess Br^−^ ions accelerate the interfacial reaction with Ag, exacerbating contact degradation. In striking contrast, further increasing the AgBr concentration introduces a high density of Ag^+^ ions within the doped layer, which effectively passivates the interface and stabilizes the Ag electrode under high bias. These findings suggest that the net stability of the Ag/MAPbBr_3_ contact is dictated by the interplay between mobile Br^−^ species (or intrinsic bromine vacancies) and the AgBr content [21,22,23,24,25].

Consequently, interfacial stability can be systematically enhanced through the dual approach of minimizing mobile Br^−^ defects and maximizing Ag^+^ incorporation. This study demonstrates a viable pathway toward achieving robust metal contacts on lead halide perovskites, with broad implications for perovskite-based optoelectronic and detection devices [6,9,26,27,28,29].

## 2. Materials and Methods

Materials: Lead bromide (PbBr_2_; 99%) and methylammonium bromide (MABr, 99%) were purchased from Sigma Aldrich (St. Louis, MO, USA). Dimethyl sulfoxide (DMSO, 99.9%) and dimethylformamide (DMF, 99.9%) were obtained from Sigma Aladdin (St. Louis, MO, USA). Metallic silver was purchased from Chinese reagent (Shanghai, China). All commercial products were used as received.

Growth of MAPbBr_3_ perovskite single crystals: High-quality MAPbBr_3_ perovskite single crystals were grown by inverse temperature crystallization [10,30]. Specifically, 1 M MABr and 1 M PbBr_2_ were dissolved in DMF solution. The solutions were filtered through a PTFE filter with a 0.22 μm pore size. The filtrate was then transferred to a culture dish, which was placed on a programmable heating station (IKA-RET con-trol-visc), and the growth temperature was 65 to 85 °C at a rate of 1 °Ch^−1^. For AgBr-doped MAPbBr_3_ single-crystal epitaxial growth, 1.5 M MABr, 1 M PbBr_2_ and 0.3 M AgBr were dissolved in DMF solution; the solutions were filtered through a PTFE filter with a 0.22 μm pore size. Then, a pristine precursor solution was mixed with AgBr-doped precursor solution with a volume ratio of 30:0 mL; 22.5:7.5 mL; 15:15 mL; 7:22 mL and 0:30 mL for 0%; 7%; 15%; 22% and 30% AgBr-doped MAPbBr_3_ epitaxial growth. Then, pristine MAPbBr_3_ single crystals were put into different precursor solutions, the growth temperature was set at 70 °C, and the growth speed was about 10 μm/min. For the metal electrodes, electrodes were deposited on the face of MAPbBr_3_ perovskite single crystals by a metal mask under a vacuum of 6 × 10^−4^ Pa.

Characterization of the MAPbBr_3_ perovskite single crystals: X-ray diffraction (XRD) patterns were obtained using an X’TRA system with a Cu target (Thermo Fisher Scientific, Ecublens, Switzerland). Optical absorption spectra were measured by UV-vis spectroscopy in the range from 300 to 2000 nm (Lab Tech Bluestar, Ortenberg, Germany). Current–voltage characteristics were measured using a Keithley 4200SC semiconductor analyzer (Keithley, Solon, OH, USA). X-ray photoelectron spectroscopy was obtained using ESCALAB Xi+ (Thermo Fisher Scientific, Ecublens, Switzerland).

## 3. Results

**Figure 1:**
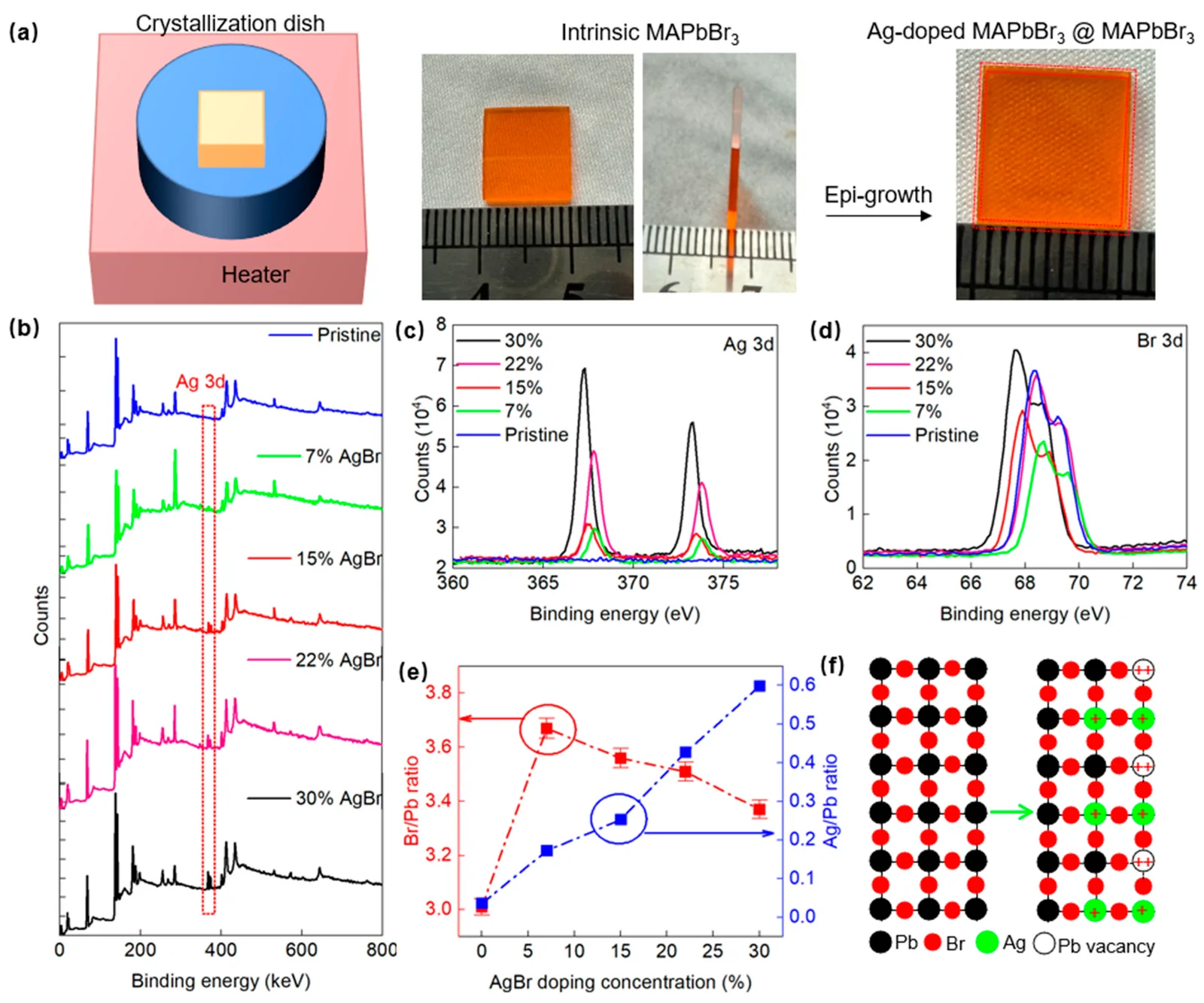
Growth and characterization of the Ag-doped MAPbBr_3_ single crystals. (**a**) Fabrication process of the Ag-doped MAPbBr_3_ single crystals. (**b**) Survey scan of MAPbBr_3_ with different AgBr doping concentrations. (**c**) Ag 3d of MAPbBr_3_ with different AgBr doping concentrations. (**d**) Br 3d of MAPbBr_3_ with different AgBr doping concentrations. (**e**) Br/Pb ratio and Ag/Pb ratio in different MAPbBr_3_ single crystals. (**f**) Schematic diagram of intrinsic MAPbBr_3_ and Ag-doped MAPbBr_3_.

As shown in Figure 1a, the MAPbBr_3_ single crystal was achieved via the inverse temperature crystallization method using a crystallization dish placed atop a heater, as shown schematically. This setup enables a slow temperature ramp (Figure S1), uniform heating and controlled solvent saturation, which are critical for promoting the formation of high-quality, large-area single crystals. The resulting intrinsic MAPbBr_3_ single crystal exhibits a uniform orange coloration and well-defined facets, indicative of low defect density and good crystallinity. Powder X-ray diffraction for pristine and AgBr-doped MAPbBr_3_ single crystals is shown in Figure S2. The weak secondary peak at a high 2θ angle was attributed to the Cu target X-ray source, which consisted of two wavelength X-ray photons (K_α1_ = 0.15405 nm and K_α2_ = 0.15443 nm). The optical transparency and sharp edges suggest minimal strain during growth for pristine and AgBr-doped MAPbBr_3_ single crystals, as shown in Figure S3. Further, an Ag-doped heterostructure was fabricated via epitaxial growth of AgBr-doped MAPbBr_3_ onto the pristine MAPbBr_3_ single crystal. The epi-growth strategy leverages the lattice matching between the substrate and the overgrown layer, minimizing interfacial defects and ensuring structural coherence. The resulting Ag-doped MAPbBr_3_@MAPbBr_3_ heterostructure retains the macroscopic morphology of the underlying crystal while incorporating Ag dopants uniformly throughout the top layer, as evidenced by the consistent color. Here, the 30% concentration has already reached the saturation point; adding more AgBr would simply fail to dissolve.

To confirm the doping concentration of the surfaces of the Ag-doped MAPbBr_3_ single crystals, the X-ray photoelectron spectroscopy (XPS) survey spectra are presented in Figure 1b. The survey spectra provide compelling evidence for the successful incorporation of Ag+ into the MAPbBr_3_ lattice across a range of doping concentrations. As illustrated by the progressive emergence and intensification of the Ag 3d core-level peak (highlighted by the red dashed box), the intensity of this characteristic signal exhibits a clear positive correlation with the nominal AgBr doping concentration. While the pristine MAPbBr_3_ single crystal sample shows no detectable Ag 3d feature, distinct peaks corresponding to Ag 3d become increasingly prominent at 7%, 15%, 22%, and 30% AgBr doping concentrations. This monotonic increase in Ag 3d intensity confirms the effective introduction of AgBr into the perovskite matrix.

High-resolution XPS spectra provide deeper insights into the chemical states and local bonding environments. As shown in Figure 1c, the Ag 3d core-level spectra exhibit characteristic doublet peaks corresponding to Ag 3d and Ag 3d, with binding energies centered around 368.0 eV and 374.0 eV, respectively. These positions are consistent with the +1 oxidation state of silver (Ag^+^), confirming that Ag substitutes for Pb^2+^ in the perovskite lattice rather than forming metallic Ag. The progressive enhancement of both peaks with increasing AgBr doping concentration further validates the successful incorporation of Ag species into the crystal structure.

Complementing the Ag 3d data, the Br 3d spectra in Figure 1d reveal subtle but meaningful changes in response to Ag doping. The Br 3d peak exhibits a slight shift toward higher binding energy and a gradual broadening with increasing Ag content. This shift can be attributed to the altered electrostatic environment around Br^−^ ions due to the replacement of divalent Pb^2+^ by monovalent Ag^+^, which reduces the overall cationic charge density and induces a slight electron withdrawal from Br. Additionally, the relative intensity of the Br 3d peak appears to decrease marginally at higher doping levels (e.g., 30%), which may reflect the formation of Pb vacancies as a charge-compensating mechanism. The Pb 4f spectra are shown in Figure S4. Upon AgBr doping, more positive holes would be induced. Thus, these holes would attract Pb 4f electrons, which causes the binding energy to decrease.

Quantitative XPS analysis (Figure 1e) reveals a linear increase in the Ag/Pb ratio with doping, whereas the Br/Pb ratio declines beyond 10% AgBr, indicating that charge neutrality is maintained not merely by Ag^+^ substitution but via the formation of Pb vacancies. This defect chemistry is visualized in Figure 1f, where Ag atoms (green) occupy Pb sites, while adjacent Pb vacancies (open circles) compensate the charge imbalance. The coexistence of these defects modifies the local electronic environment—evidenced by the Br 3d shift—and introduces shallow acceptor states that are critical for tuning carrier concentration.

**Figure 2:**
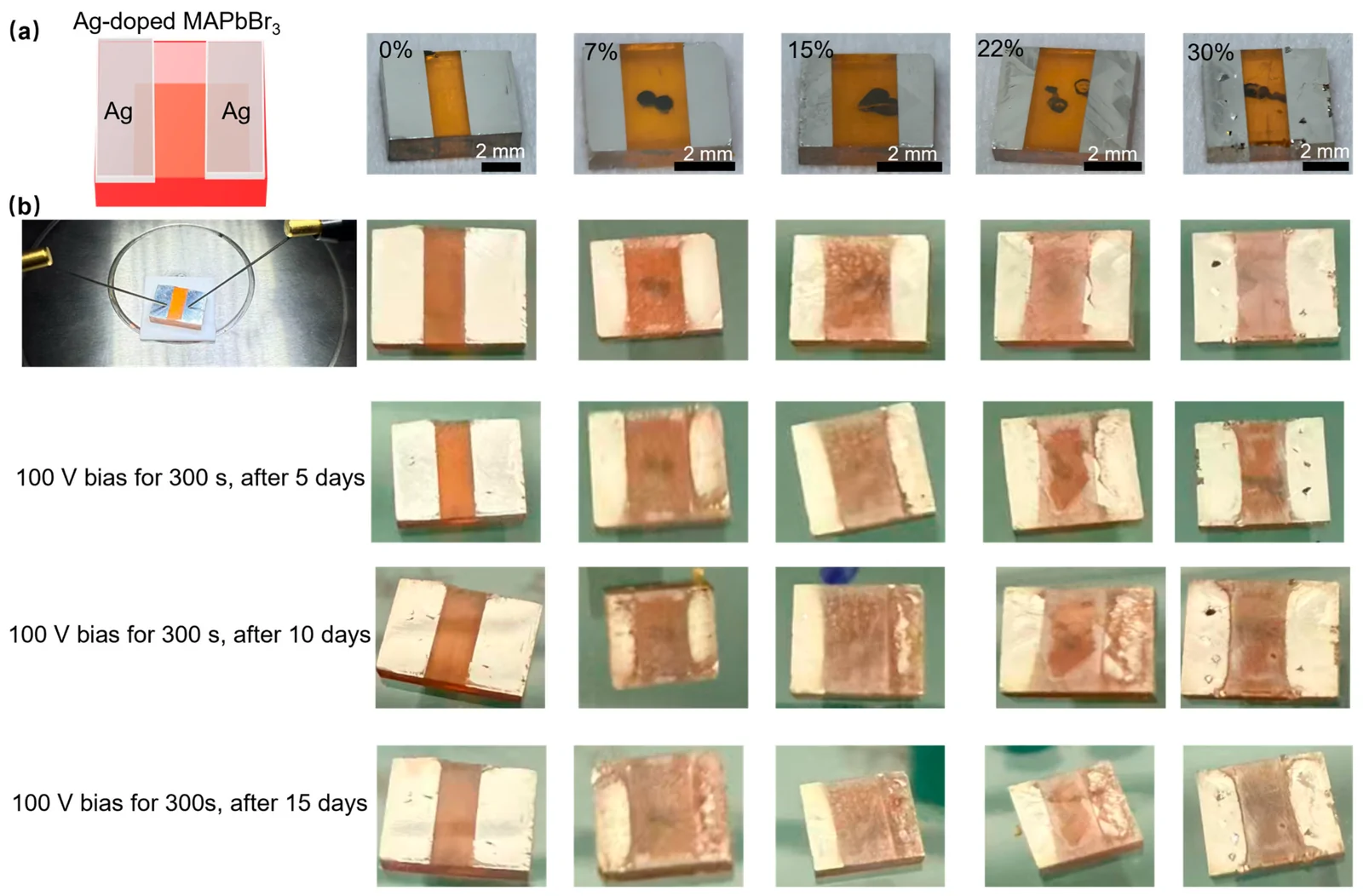
Stability of the Ag electrodes deposited on Ag-doped MAPbBr_3_ single crystals. (**a**) Structure of the tested Ag electrodes on Ag-doped MAPbBr_3_ single crystal. (**b**) Optical photos of the devices after 100 V bias for different times.

Building upon the successful incorporation of AgBr into MAPbBr_3_ single crystals, Figure 2a presents macroscopic photographs of the as-grown series. The crystals exhibit excellent morphological uniformity and well-defined facets. Notably, distinct dark marks on the back surfaces serve as physical identifiers for orientation tracking and sample indexing in subsequent characterizations. Two Ag electrodes with an active area (2 × 8^−10^ mm^2^) were deposited on the MAPbBr_3_ single crystals.

Electrical aging tests under prolonged bias reveal a striking concentration-dependent stability trend (Figure 2b). For the first step, a 100 V bias was applied on the electrodes for 300 s, then the devices were stored in ambient air for 5 days. For the second step, after 5 days of storage in ambient air, the devices were biased at 100 V for 300 s, and photos were taken. Further, in the next steps, we just repeated the 100 V bias for 300 s, then stored it in ambient air for 5 days. We found that the Ag electrodes could not be observed by the eye. As expected from the incorporation of Ag^+^ ions, AgBr doping significantly reduces the resistivity of MAPbBr_3_, resulting in a markedly higher baseline current compared to the pristine crystal. However, the long-term operational stability exhibits a non-monotonic dependence on doping concentration. Macroscopic inspection reveals that Ag electrodes on the 15% Ag-doped sample suffer severe degradation under bias, whereas those on the pristine crystal remain relatively intact. In contrast, the 30% Ag-doped sample demonstrates exceptional robustness, retaining a stable polarization response over time compared to lower-doped counterparts (7–22%). This enhanced endurance is attributed to the saturated incorporation of Ag^+^, which effectively “pins” the lattice and suppresses electric-field-induced ion migration. These results identify 30% as the optimal doping threshold, where maximized defect occupancy transitions from a liability into a stabilizing factor against electrical stress. Additionally, we can observe the surface becoming opaque and developing a white, mist-like substance. We think this layer could be AgBr.

**Figure 3:**
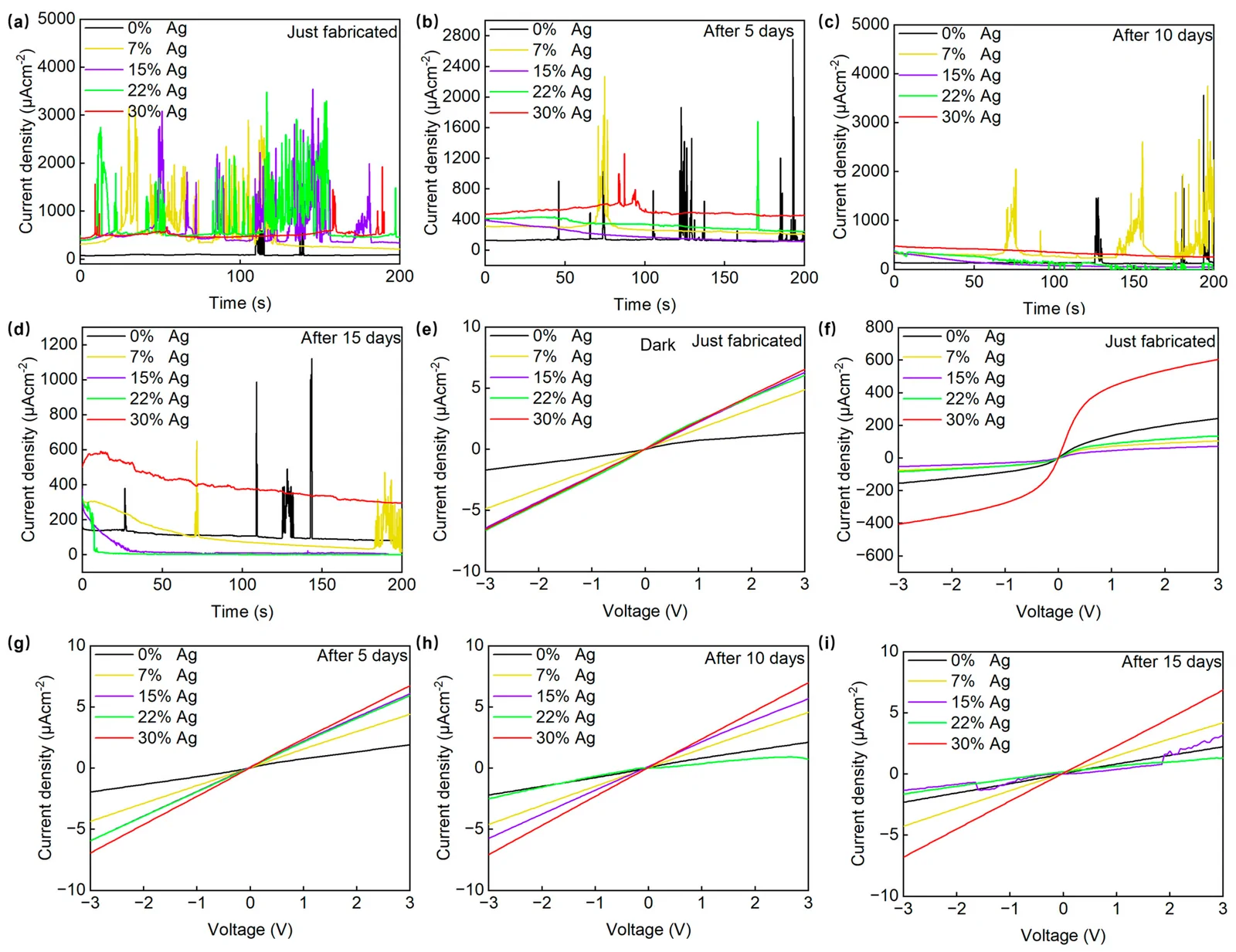
I-V and I-T characterization of the Ag electrodes deposited on Ag-doped MAPbBr_3_ single crystals. (**a**) I-T characterization with 100 V bias, just fabricated. (**b**) I-T characterization with 100 V bias, after 5 days. (**c**) I-T characterization with 100 V bias, after 10 days. (**d**) I-T characterization with 100 V bias, after 15 days. (**e**) −3 to 3V I-V characterization in the dark, just fabricated. (**f**) −3 to 3V I-V characterization under 1 mWcm^−2^ light, just fabricated. (**g**) −3 to 3V I-V characterization in the dark, after 5 days. (**h**) −3 to 3V I-V characterization in the dark, after 10 days. (**i**) −3 to 3V I-V characterization in the dark, after 15 days.

To elucidate the failure mechanisms of Ag electrodes, time-dependent current–voltage (I–V) and current–time (I–T) measurements were conducted. As shown in Figure 3a, freshly fabricated Ag electrodes fail to establish a stable metal–semiconductor contact under high bias. This instability is particularly acute for the 7–22% Ag-doped series, where numerous current spikes are observed, indicative of aggressive electrochemical corrosion at the Ag/MAPbBr_3_ interface. In contrast, both the 30% Ag-doped and pristine MAPbBr_3_ devices exhibit only sporadic spikes, suggesting a significantly milder electrochemical reaction.

After 5 days of aging (Figure 3b), the current in the 7–22% doped crystals decreased markedly due to progressive electrode degradation. Conversely, the 30% Ag-doped sample retained a stable dark current, reflecting its superior interfacial robustness. By day 10 (Figure 3c) and day 15 (Figure 3d), the Ag electrodes in the 7–22% doped crystals had completely failed, yielding the lowest current under 100 V bias. Notably, the 30% Ag-doped MAPbBr_3_ exhibited no current spikes, confirming the formation of a stable Ag/MAPbBr_3_ contact. In contrast, the pristine crystal continued to display sporadic spikes, suggesting that while stable, it lacks the optimized interfacial passivation afforded by the optimal Ag concentration.

Complementary I–V characterization further elucidates the interfacial properties (Figure 3e–i). As shown in Figure 3e, the 30% Ag-doped MAPbBr_3_ exhibits the lowest contact resistance. The linear I–V characteristics observed for the Ag/30% Ag-doped MAPbBr_3_ interface confirm the formation of an ohmic contact, which mitigates localized electric field enhancement and suppresses electrochemical corrosion. Under illumination (Figure 3f), the 30% Ag-doped device yields the highest photocurrent.

Long-term I–V stability is summarized in Figure 3g–i. While both the pristine and 30% Ag-doped devices maintain stable I–V profiles over 15 days, the 7–22% doped series exhibits a continuous decline in current, consistent with the progressive electrode failure observed in the I–T measurements. The current-density decreases during I-T characterization were attributed to Br-ion migration under bias.

## 4. Discussion

**Figure 4:**
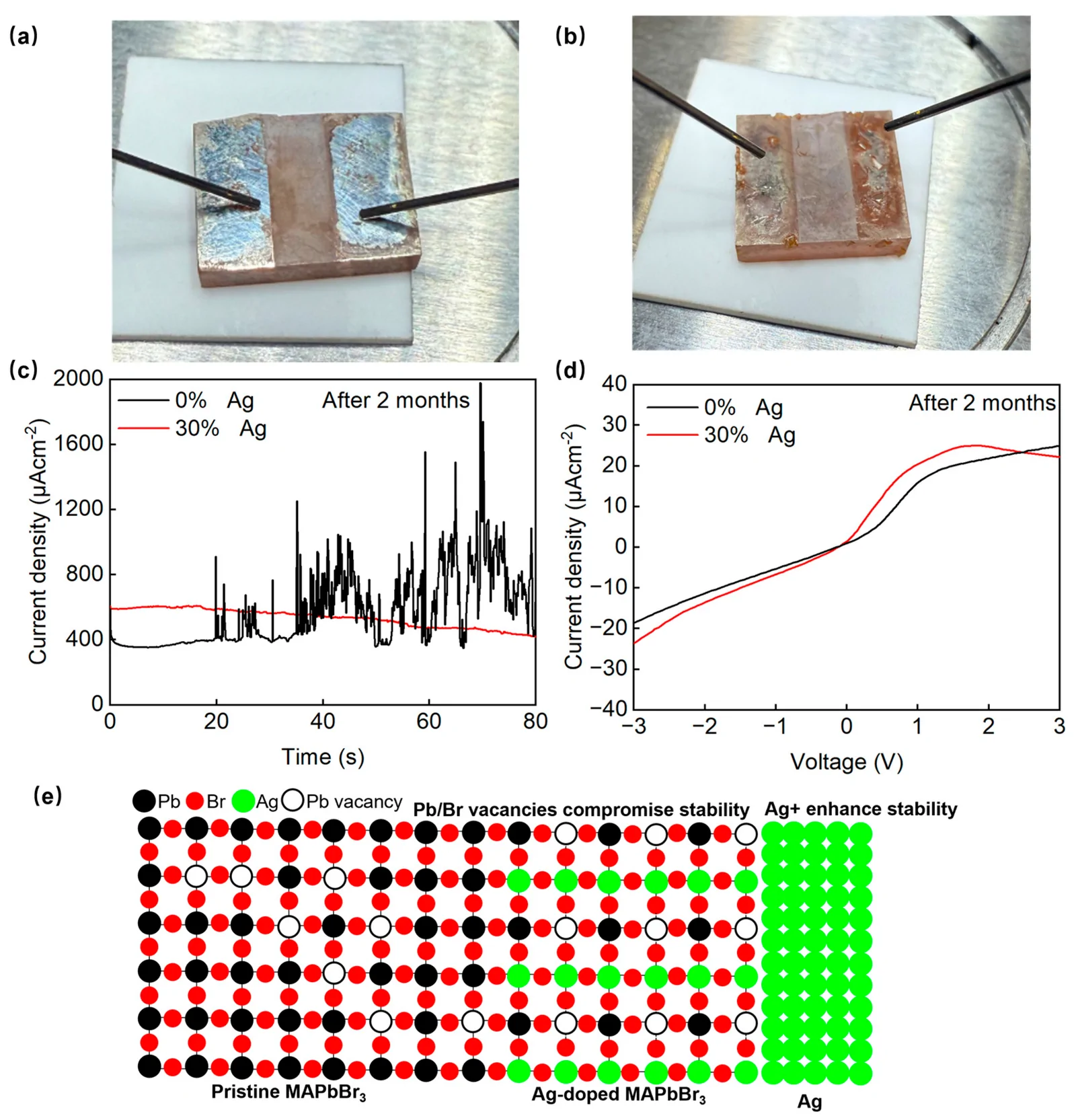
Ag electrodes deposited on pristine and 30% Ag-doped MAPbBr_3_ single crystals after 2 months. (**a**) Optical photo of pristine MAPbBr_3_ after 2 months. (**b**) Optical photo of 30% Ag-doped MAPbBr_3_ after 2 months. (**c**) I-T characterization under 100 V bias after 2 months. (**d**) I-V characterization after 2 months. (**e**) Schematic diagram of the stability influence, including Pb/Br vacancies and AgBr dopant.

After a total of 15 days, the Ag electrodes were destroyed for 7%, 15% and 22% AgBr-doped MAPbBr_3_ single crystals. Thus, pristine and 30% AgBr-doped MAPbBr_3_ single crystals were stored in ambient air for 45 days. To further assess the long-term stability, the aging study was extended to two months. As shown in Figure 4a, the pristine MAPbBr_3_ single crystal exhibits spontaneous chemical degradation, wherein metallic Ag reacts with the perovskite matrix, even in the absence of an external bias. This results in the oxidation of Ag and the subsequent diffusion of Ag^+^ into the lattice. In contrast, the 30% Ag-doped crystal (Figure 4b) also displays distinct surface degradation; however, the morphological evolution differs significantly from that of the pristine sample, suggesting that the pre-incorporated Ag^+^ alters the degradation pathway.

This visual assessment is quantitatively corroborated by electrical measurements. The current density–time (J–t) curve (Figure 4c) reveals that the pristine (0% Ag) device suffers from severe fluctuations and noise, indicative of unstable conductive filament dynamics. Conversely, the 30% Ag-doped device maintains a steady current density of approximately 500–600 μA cm^−2^, with negligible deviation from its initial state, confirming its exceptional operational stability. Furthermore, the J–V characteristics (Figure 4d) demonstrate that the 30% Ag device exhibits a higher current density response across the entire voltage sweep compared to the pristine device. This enhancement suggests that optimal Ag doping not only stabilizes the interface but also improves the overall electrical conductivity and reliability.

Consequently, the stability of the Ag/MAPbBr_3_ contact is governed by a delicate interplay between two critical factors (Figure 4e): the concentration of Pb/Br vacancies and the Ag doping level. While vacancies facilitate ion migration and corrosion, a saturated Ag^+^ concentration effectively suppresses these deleterious processes, thereby transforming a potential liability into a stabilizing feature. This halide migration phenomenon could provide useful information for perovskites applied in other approaches.

## 5. Conclusions

In this work, the stability of Ag electrodes deposited on AgBr-doped MAPbBr_3_ single crystals was systematically investigated. The incorporation of AgBr introduces additional Br^−^ ions, Pb vacancies, and free holes into the lattice. At low doping concentrations, the excess Br^−^ promotes electrode instability through aggressive electrochemical corrosion. Conversely, a high concentration of AgBr dopants significantly enhances the interfacial robustness, leading to stable electrode performance. We attribute this counterintuitive trend to the saturated incorporation of Ag^+^, which effectively immobilizes mobile halide ions and suppresses their migration to the interface. These findings elucidate the critical role of metal halide doping in governing ion migration and provide valuable insights for achieving stable metal–perovskite contacts in optoelectronic devices [31].

## Data Availability

The original contributions presented in this study are included in the article and Supplementary Materials. Further inquiries can be directed to the corresponding author.

## Supplementary Materials

The following supporting information can be downloaded at: https://www.mdpi.com/article/10.3390/nano16211379/s1, Figure S1: Full growth profile for growth of MAPbBr_3_ single crystal. Figure S2: X-ray diffraction result of pristine and AgBr-doped MAPbBr_3_ single crystals. Figure S3: Absorption curve of pristine and AgBr-doped MAPbBr_3_ single crystals. Figure S4. Pb 4f of MAPbBr_3_ with different AgBr doping concentration.
