# Supplementary material for: Enhancing the Stability of Ag/CH3NH3PbBr3 Interfaces via AgBr Doping
Source: Nanomaterials (Basel). 2026 Oct 28;16(21):1379. doi: 10.3390/nano16211379 (PMC13765350; doi:10.3390/nano16211379)
Supplement: Supplementary file 1 [file nanomaterials-16-01379-s001.zip › nanomaterials-4608342-supplementary.pdf]

# Supporting information for " Enhancing the Stability of Ag/ $CH_3NH_3PbBr_3$ Interfaces via AgBr Doping"

**Chao Wang[1,2,3], Aiwu Yu [4], Yuzhu Pan [5], Yulian Zhang[1,2], jinbao Chen[1,2], Xin Wang[1,2*]**

1 National Key Laboratory of Aerospace Mechanism, Nanjing University of Aeronautics and Astronautics, Nanjing 210016, China.
2 College of Physics, Nanjing University of Aeronautics and Astronautics, Nanjing 210016, China.
3 Shanghai Institute of Spacecraft Equipment, Shanghai, 200000, China
4 Shanghai Aerospace Equipments Manufacturer Co.Ltd，Shanghai 200245，China;
5 Key Laboratory of Semiconductor Display Materials and Chips, Suzhou Institute of Nano-Tech and Nano-Bionics, Chinese Academy of Sciences, Suzhou 215123, China
* Correspondence: xin-wang@nuaa.edu.cn

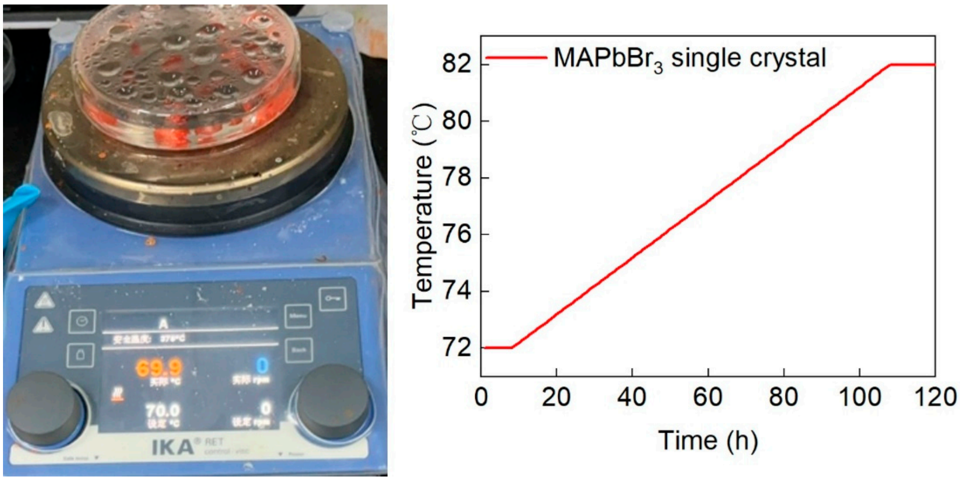


Figure S1. Full growth profile for growth of $MAPbBr_3$ single crystal.

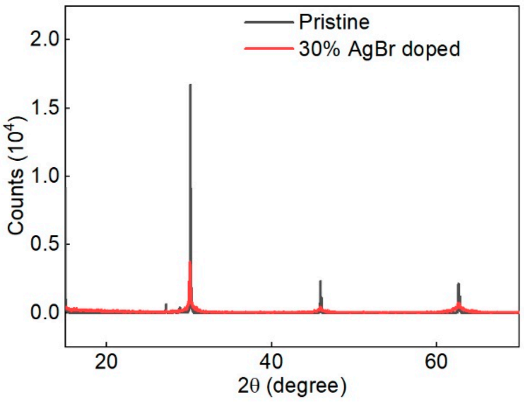


Figure S2. X-ray diffraction result of pristine and AgBr-doped $MAPbBr_3$ single crystal

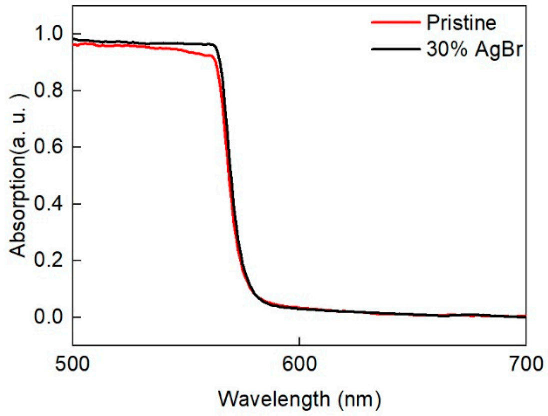


Figure S3. Absorption curve of pristine and AgBr-doped $MAPbBr_3$ single crystal

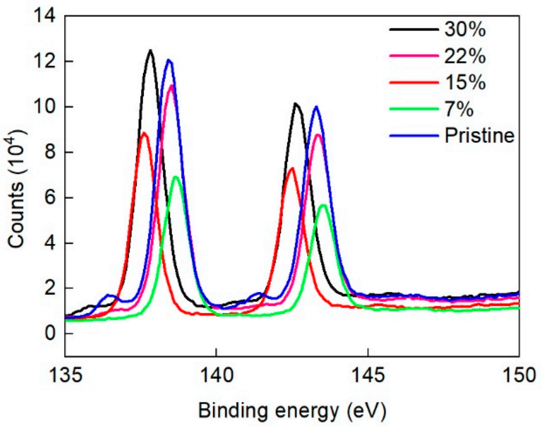


Figure S4. Pb 4f of $MAPbBr_3$ with different AgBr doping concentration.
